# Expansion of airway basal epithelial cells from primary human non‐small cell lung cancer tumors

**DOI:** 10.1002/ijc.31383

**Published:** 2018-04-06

**Authors:** Robert E. Hynds, Assma Ben Aissa, Kate H.C. Gowers, Thomas B.K. Watkins, Leticia Bosshard‐Carter, Andrew J. Rowan, Selvaraju Veeriah, Gareth A. Wilson, Sergio A. Quezada, Charles Swanton, on behalf of the TRACERx Consortium, Sam M. Janes

**Affiliations:** ^1^ UCL Cancer Institute, CRUK Lung Cancer Centre of Excellence University College London London United Kingdom; ^2^ Translational Cancer Therapeutics Laboratory The Francis Crick Institute London United Kingdom; ^3^ Cancer Immunology Unit UCL Cancer Institute, University College London London United Kingdom; ^4^ Lungs for Living Research Centre, UCL Respiratory University College London London United Kingdom

**Keywords:** lung cancer, cell culture, epithelial cells, basal cells, stem/progenitor cells

## Abstract

Pre‐clinical non‐small cell lung cancer (NSCLC) models are poorly representative of the considerable inter‐ and intra‐tumor heterogeneity of the disease in patients. Primary cell‐based *in vitro* models of NSCLC are therefore desirable for novel therapy development and personalized cancer medicine. Methods have been described to generate rapidly proliferating epithelial cell cultures from multiple human epithelia using 3T3‐J2 feeder cell culture in the presence of Y‐27632, a RHO‐associated protein kinase (ROCK) inhibitor, in what are known as “conditional reprograming conditions” (CRC) or 3T3 + Y. In some cancer studies, variations of this methodology have allowed primary tumor cell expansion across a number of cancer types but other studies have demonstrated the preferential expansion of normal epithelial cells from tumors in such conditions. Here, we report our experience regarding the derivation of primary NSCLC cell cultures from 12 lung adenocarcinoma patients enrolled in the Tracking Cancer Evolution through Therapy (TRACERx) clinical study and discuss these in the context of improving the success rate for *in vitro* cultivation of cells from NSCLC tumors.

## Abstract 2

**What's new?**

Traditionally, the primary culture of human cancer cells has been challenging. A new protocol known as 3T3 + Y has allowed cancer cell cultures to be established across multiple cancer types, but reports are inconsistent for non‐small cell lung cancer (NSCLC). Here, the authors sought to expand tumor cells from surgically resected primary human lung adenocarcinoma tumors within the TRACERx clinical study using 3T3 + Y. Contaminating normal epithelial cells were the predominant cell type in early passage cultures, although *KRAS*‐mutant tumor cells were cultured in one donor culture. The researchers urge caution in the use of NSCLC primary cell cultures and call for further investigation in 3T3 + Y applications.

## Introduction

Progress in normal epithelial biology has shown that the addition of a RHO‐associated protein kinase (ROCK) inhibitor^1^, ^2^ to traditional keratinocyte growth medium and mitotically inactivated 3T3‐J2 murine embryonic feeder cell co‐culture^3^ can increase the number of cells expanded *in vitro* and help to initiate cultures from small samples. Traditionally, the primary culture of human cancer cells has been challenging, with few tumors amenable to culture on plastic, so this protocol, known as “conditional reprogramming” or “3T3 + Y,” has naturally attracted attention in the cancer community. To date, variants of this protocol have allowed cancer cell cultures to be established across multiple cancer types including lung, prostate, pancreas and colon.^4^, ^5^, ^6^

In non‐small cell lung cancer (NSCLC), a number of reports demonstrate successful primary tumor cell culture using fibroblast co‐culture and ROCK inhibition.^7^, ^8^, ^9^, ^10^ However, others have found that normal epithelial cells are preferentially expanded in these conditions.^11^, ^12^ For example, Sette *et al*. recently showed strong selection for normal, non‐tumorigenic airway epithelial cells and/or no tumor cell expansion from 19 surgically resected NSCLC tumors of mixed histological subtypes, 4 pulmonary metastases, 2 lung cancer brain metastases and 1 patient‐derived xenograft (PDX) model.^13^

Here, we present cell culture outcomes for 12 lung adenocarcinoma patients enrolled in the on‐going Tracking Cancer Evolution through Therapy (TRACERx) clinical study.^14^, ^15^ We found that contaminating normal epithelial cells were the predominant cell type present in early passage cultures, although tumor cells were cultured in one case. We discuss these findings with a view to improving the efficacy of primary cell culture methodologies for early stage NSCLC tumors.

## Materials and Methods

### Cell culture

Ethical approval for generating patient‐derived models was obtained through the Tracking Cancer Evolution through Therapy (TRACERx) clinical study (REC reference 13/LO/1546). 3T3‐J2 mouse embryonic fibroblast feeder cells were cultured in DMEM (Invitrogen, UK) containing 1X penicillin/streptomycin (Gibco, UK) and 9% bovine serum (Invitrogen). Feeder layers were prepared by inactivating confluent 3T3‐J2 fibroblasts in medium containing 0.4 μg/ml mitomycin C (Sigma Aldrich, UK) for 2 hr and re‐plating these at 20,000 cell/cm^2^ in fibroblast growth medium. Feeder cells were allowed to adhere overnight before tumor‐derived cells were added. Tumors were received on ice in transport medium consisting of MEM alpha medium (Gibco) containing 1X penicillin/streptomycin (Gibco), 1X gentamicin (Gibco) and 1X amphotericin B (Fisher Scientific, UK) after fresh dissection. Tumors were digested to single cell suspensions in serum‐free RPMI medium (Gibco) containing 0.25 U/ml Liberase^TM^ (Roche, UK) and 0.1 mg/ml DNAse I (Sigma) in a waterbath at 37°C for 2 hr with regular agitation. Tumor‐derived cells were plated on 3T3‐J2 feeder layers in epithelial cell culture medium containing EGF and Y‐27632 (3T3 + Y) as previously described.^16^, ^17^ Airway spheroids, or “tracheospheres,” were generated using a previously published protocol.^16^

### Histology

Samples were fixed in 4% PFA for 30 min at room temperature (tracheospheres^16^) or overnight at 4°C (subcutaneous tumors and primary tissue) and processed for paraffin embedding according to standard protocols. 4 μm formalin‐fixed, paraffin‐embedded (FFPE) sections were hematoxylin and eosin (H&E) stained using an automated tissue processor (Tissue‐Tek, The Netherlands). 40× images were acquired using a NanoZoomer 2.0HT whole slide imaging system (Hamamatsu Photonics, Japan). For immunofluorescence, slides were dewaxed using an automated protocol and antigen retrieval was performed using citrate buffer (pH 6.0). Primary antibodies were anti‐MUC5B (Sigma HPA008246), anti‐ACT (Sigma T6793) and anti‐p63 (Abcam, UK, ab53039). Species‐appropriate secondary antibodies were conjugated to Alexa Fluor dyes (Molecular Probes, UK; 1:500). Images were acquired using a Zeiss LSM700 confocal microscope.

### Immunocytochemistry

Culture‐expanded cells were seeded into 8‐well, collagen‐coated chamber slides (Ibidi, Germany) with feeder cells and expanded for 4 days. Cells were washed with PBS and fixed with 4% PFA at room temperature for 30 min. Cells were stored at 4°C in PBS until staining. Cells were permeabilized and blocked for 1 hr in PBS containing 10% FBS and 0.01% Triton X‐100 (Fisher Scientific). Primary antibodies were anti‐pan‐keratin (Dako, USA, M3515; 1:500), anti‐keratin 5 (Abcam ab52635; 1:200), anti‐p63 (Abcam ab124762; 1:300) and anti‐TTF‐1 (Abcam ab76013; 1:200). These were incubated overnight at 4°C in block buffer without Triton X‐100. Cells were washed three times with PBS before addition of species‐appropriate secondary antibodies (Alexa Fluor; Molecular Probes; 1:500) at room temperature for 2 hr in block buffer without Triton X‐100.

### Subcutaneous xenografts

Animal studies were approved by the University College London Biological Services Ethical Review Committee and licensed under UK Home Office regulations. Six to eight week‐old male immunocompromised NOD/SCID/gamma (NSG) mice were purchased from Charles River, kept in individually ventilated cages under specific pathogen‐free conditions and had *ad libitum* access to both sterile food and autoclaved water. To generate subcutaneous tumors, mice were anaesthetized using 2–4% isoflurane, the right flank was shaved and cleaned before 200 μl growth‐factor reduced Matrigel containing 1 × 10^6^ cultured cells was injected subcutaneously. Animals were observed during recovery, then regularly monitored for tumor growth. Experiments lasted for 3 months or were terminated before tumors reached 1.5 cm^3^ in volume.

### Next‐generation sequencing (NGS)

NGS of a TruSeq custom amplicon for lung cancer panel that comprises 107 hotspot amplicons from 15 genes was performed using the MiSeq system (Illumina). The NGS amplicon library preparation was performed using 125 ng DNA as input for patient tissue and cell cultures derived from patient‐matched tumors. The resulting sequence library was normalized and pooled prior to sequencing on a MiSeq instrument according to the manufacturer's instructions (Illumina, USA). We used a MiSeq Reagent Kit v2 (300 cycles) with 2 × 150 paired‐end sequencing design according to the manufacturer's instructions (Illumina). The human hg19 genome assembly was used to align the paired‐end raw reads.

The variant allele frequencies of 24 SNPs previously identified by Pengelly *et al*.^18^ were extracted for each sequenced cell culture and compared to previously sequenced tumor regions from the same patient to confirm that no sample swaps had occurred between patients.

SAMtools mpileup^19^ (0.1.19; base phred score >20 and read mapping quality >20, BAQ computation disabled), Varscan2^20^ (v2.4.1; includes running fpfilter.pl) and MuTect^21^ (v1.1.7) were used to locate non‐reference positions in cell culture, tumor and germline samples. To reduce false positive variant calls, additional filtering was performed. A variant allele frequency of 5% was required with somatic *p*‐values <=0.01, sequencing depth in each region was required to be >=30 and >=10 sequence reads had to support the variant call. In contrast, the VAF in the germline data had to be <=1% whilst the number of reads supporting the variant had to be <5. Finally, variants designated as “germline” by VarScan2 from all regions in all 100 patients within the TRACERx exome cohort^14^ were combined so that every germline variant detected in the cohort had an associated TRACERx population frequency. SNVs were filtered if they were found to have >1% frequency in the TRACERx germline cohort.

## Results

We attempted cell culture from 12 patients (median age = 75 years, range = 58–90 years), whose surgically resected primary lung adenocarcinoma (LUAD) tumors (stage II [*n* = 9] or III [*n* = 3]) are being analyzed in the on‐going Tracking Cancer Evolution through Therapy (TRACERx) clinical study,^14^, ^15^ using a published protocol.^17^ We obtained primary epithelial cell cultures that could be passaged from 10 (83.3%) of these.

**Figure 1:**
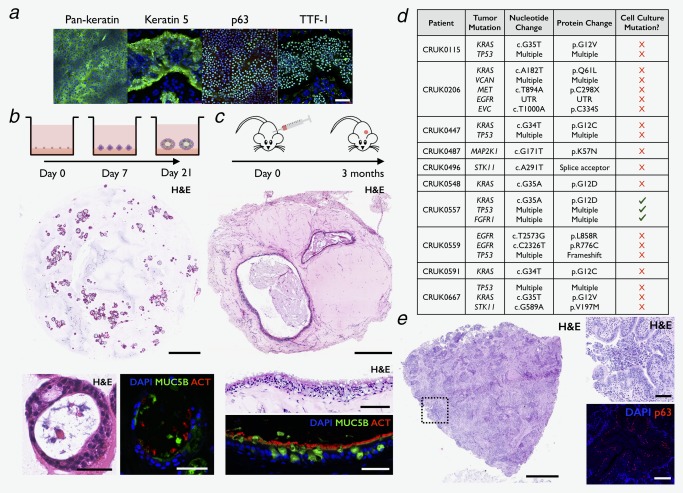
Frequent expansion of normal human airway epithelial cells from non‐small cell lung cancer (NSCLC) tumors in 3T3‐J2 co‐culture in the presence of Y‐27632 (3T3 + Y). (*a*) Immunofluorescence staining for pan‐keratin, keratin 5, p63 and TTF‐1 in a representative culture (*n* = 3 patients; scale bar = 100 μm). (*b*) *In vitro* tracheosphere assay. Hematoxylin and eosin (H&E) staining (top panel, scale bar = 1 mm; bottom left panel, scale bar = 50 μm) demonstrated airway differentiation capacity of cell cultures expanded from NSCLC tumors (<passage 5; representative images, *n* = 4 patients). Immunofluorescence confirmed the presence of ACT+ ciliated cells and MUC5B+ mucosecretory cells (bottom right panel, scale bar = 50 μm). (*c*) Subcutaneous injection tumorigenesis model. Injection of cultured cells (<passage 5) into NSG mice did not lead to tumor formation but H&E analysis showed the presence of epithelium resembling that found in human airways after 3 months (3/3 patients; top panel scale bar = 500 μm; middle panel scale bar = 50 μm). Immunofluorescence confirmed the presence of ACT+ ciliated cells and MUC5B+ mucosecretory cells (bottom panel, scale bar = 50 μm). (*d*) Next‐generation sequencing using the Illumina MiSeq platform did not detect mutations found in the tumor of origin in 9/10 NSCLC cell cultures. “Multiple” indicates that more than one variant results from the specific mutation detected, i.e., it affects multiple isoforms at different positions. (*e*) H&E analysis showed bronchiolar epithelium within a human lung adenocarcinoma tumor (left panel, scale bar = 1 mm; top right panel, scale bar = 100 μm). Immunofluorescence confirmed the presence of p63+ basal epithelial cells within these bronchioles (bottom right panel, scale bar = 50 μm). [Color figure can be viewed at http://wileyonlinelibrary.com]

Tumor‐derived cells were morphologically indistinguishable from normal human airway epithelial cells in 3T3 + Y culture and expressed proteins associated with airway basal cells, such as keratin 5 and the transcription factor p63 (Fig. 1 *a*). The LUAD‐associated protein TTF‐1 (also known as homeobox protein NKX‐2.1) was expressed by cultured cells (Fig. 1 *a*; 3/3) but is also expressed by normal airway basal cells. To morphologically distinguish contaminating basal cells from tumor cells, we grew four of the cell cultures in 3D differentiation conditions,^16^ expecting that cells would form either hollow normal airway “tracheospheres” or solid tumor spheroids.^22^ Cells underwent lumen formation and generated epithelial spheroids containing differentiated airway epithelial cell types, suggesting that we had expanded normal airway basal cells from these tumors (Fig. 1 *b*; 4/4).

To further examine whether we had expanded cancer cells or normal epithelial cells, we subcutaneously injected 1 × 10^6^ culture‐expanded cells in 100% growth factor‐reduced Matrigel in immunocompromised (NSG) mice. After 3 months, no tumors developed in mice injected with tumor‐derived epithelial cell cultures (0/3). In Matrigel retrieved from these mice, we found epithelial cells with the characteristics of differentiated normal human proximal airway epithelial cells (Fig. 1 *c*).

**Figure 2:**
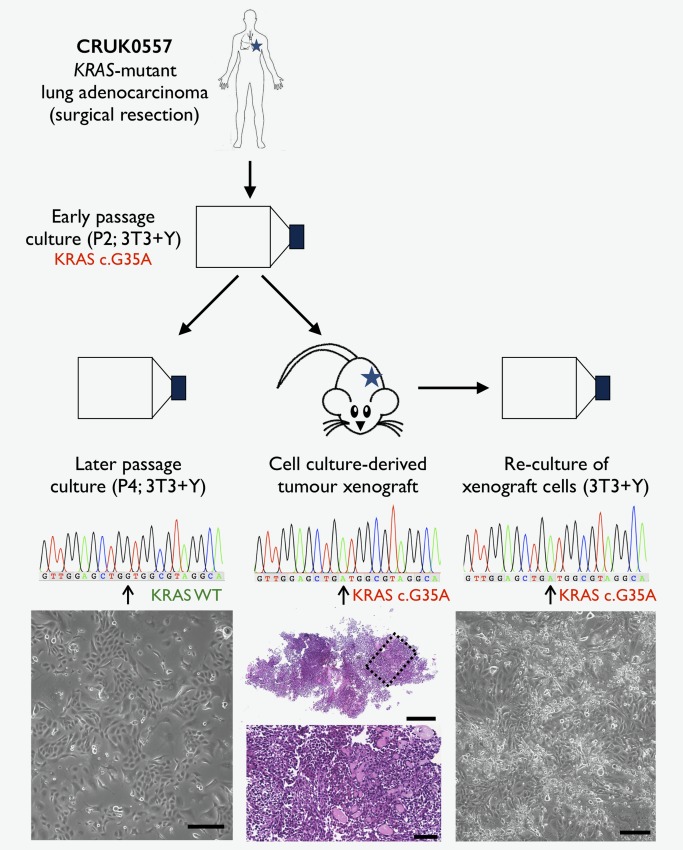
Expansion of primary human tumor cells from a *KRAS*‐mutant lung adenocarcinoma in 3T3‐J2 co‐culture in the presence of Y‐27632 (3T3 + Y). Cancer mutation‐bearing cells were detected in 1 of our 10 early passage patient cultures (CRUK0557) by next‐generation sequencing. Sanger sequencing of the same cell culture at later passage (P4; scale bar = 100 μm) revealed that the *KRAS* mutation was no longer detected (left panel). Injection of the early passage (P2) cell culture into an immune‐compromised (NSG) mouse generated a tumor with mutant *KRAS* (center panel). A hematoxylin and eosin (H&E)‐stained section is shown (scale bar = 500 μm). A magnified view of the black dotted box is shown below (scale bar = 100 μm). Re‐culture of cells from the cell culture‐derived xenograft in 3T3+Y was possible (right panel; scale bar = 100 μm) and mutant *KRAS* was again detected by Sanger sequencing. [Color figure can be viewed at http://wileyonlinelibrary.com]

The preferential expansion of normal human airway basal cells in these conditions was subsequently confirmed using sequencing approaches. The primary patient tumors had undergone next‐generation sequencing (NGS) of a targeted panel of SNVs in 15 lung cancer‐associated genes and amplifications in a further 3 genes using the Illumina MiSeq system within the TRACERx study. Equivalent NGS of the ten patient‐matched early passage cell cultures confirmed that cancer mutations were not detected in nine of ten cultures (≤5 passages; Fig. 1 *d*). Since p63+ cells can be found within LUAD tumors (Fig. 1 *e*), it is likely to be these cells that are expanded in culture. However, in one passage 2 cell culture that was not investigated in prior assays, we detected all three mutations that were found in the patient's tumor at high variant allele frequencies by MiSeq (81.3% mutant *KRAS*; Fig. 1 *d*) and these cells gave rise to a tumor on subcutaneous injection in an NSG mouse (Fig. 2). Cells could be re‐cultured in 3T3 + Y following recovery of the xenograft tumor and Sanger sequencing demonstrated that these were *KRAS* mutant (Fig. 2). Interestingly, Sanger sequencing of the parent cell culture just two passages later (i.e., passage 4) did not detect mutant *KRAS* (Fig. 2), suggesting that normal epithelial cells rapidly out‐grow cancer cells in this culture system when both are present.

Overall, these data show that 3T3 + Y conditions supported tumor cell expansion for just 1 of 10 NSCLC tumors and that selection of cancer cells over normal epithelial cells is essential for cancer cell culture maintenance.

## Discussion

Our results suggest that a very small number of contaminating normal airway basal cells contained within LUAD tumors are sufficient to initiate cell cultures in these conditions, corroborating the recent findings of Sette *et al*.^13^ and others.^11^, ^12^ Even after careful dissection of tissue by a pathologist to obtain normal lung and tumor samples, bronchioles containing normal airway epithelium can be found in the NSCLC tumor microenvironment (Fig. 1 *e*).

It has been possible to establish cultures of tumor mutation‐bearing cells in many NSCLC cases,^8^, ^9^, ^23^ including from PDX tumors,^24^ but the widespread utility of the “conditional reprogramming” method still remains to be established, particularly in early stage disease and from tumor samples derived from surgical resection or autopsy. Studies that report normal epithelial cell expansion have tended to initiate culture from early‐stage primary NSCLC tumors, whereas studies in which cancer cells have been expanded have initiated culture using biopsies or effusions from patients with late‐stage, therapy‐resistant disease^7^, ^8^, ^9^ or from PDX models,^24^ which may be more aggressive and/or more adaptable to cell culture. This would be consistent with traditional NSCLC cell culture methods and PDX models, where patient models are more readily established from late‐stage, metastatic or therapy‐resistant disease than from early‐stage primary disease. As well as disease stage, tumor genotype could also be an important determinant of culture success in these conditions: previous studies focus on biopsies and plural effusions from *EGFR*‐mutant/*ALK*‐positive tumors, while 7 of our 10 LUAD tumors were driven by mutant *KRAS*.

Despite containing less tissue, biopsies or pleural effusion samples might also be advantageous for initiating tumor cultures by virtue of their lack of contaminating normal epithelium^9^ as normal epithelial cells outgrow patient‐matched cancer cells in controlled 3T3 + Y experiments.^11^ Normal cells also outcompete tumor‐derived cells during cancer organoid derivation. Here, it has been possible to develop tumor‐specific media to select tumor organoids over normal colonic^25^ and hepatic^26^ epithelial organoids. For example, intestinal epithelial cells are dependent on exogenous Wnt in culture but constitutively active Wnt signaling, due to genomic aberrations in genes such as *APC* and/or *CTNNB1*, often confers tumor cells with the ability to survive culture in Wnt‐free culture media. Such strategies might be relevant in NSCLC culture: for example, EGF could be withdrawn in cases where tumors have *EGFR*‐activating mutations or nutlin‐3 could be added to *P53*‐mutant tumor cultures.^25^ These selection strategies could be effective in cases where the tumor genotype is known at the time of surgery or in lung squamous cell carcinoma cell culture where the vast majority of tumors are *P53* mutant. While the current data suggest LUAD tumor cells are lost very early during culture in 3T3 + Y, it remains possible that normal cells actively limit tumor cell expansion and selection might permit the growth of tumor cells.

Protocol optimization is clearly required to adapt recent progress in epithelial biology towards routine utility in cancer studies. Protocol differences exist between the aforementioned studies: key studies have used inactivated human dermal fibroblasts^7^, ^8^ as feeder layers rather than the mouse embryonic fibroblasts often used in those that see normal cell expansion and it has also been possible to transition tumor cell cultures off feeder layers after 6 months of culture establishment.^8^ We have previously demonstrated that adult human lung fibroblasts do not support long‐term normal airway epithelial cell expansion in the same way as 3T3‐J2 cells^17^ so the use of adult and/or human feeder cells might favor tumor cell expansion. Relevant biological differences might also exist between mitotic inactivation using irradiation^4^, ^9^ rather than mitomycin C, as used here. Alternatively, different tissue acquisition methods,^9^ feeder cell‐conditioned medium,^10^, ^27^ low oxygen,^10^ harsh tissue digestion protocols^26^ and/or initially serum‐starving cells^6^ might help to select tumor cells by inducing differentiation or apoptosis in normal epithelial cells.

In summary, researchers should remain cautious in their use of NSCLC primary cell cultures, particularly from early‐stage tumors; genomic analyses should confirm the tumor origin of individual cultures and, if present, the proportion of tumor cells should not be assumed to be consistent over passage.^23^ While 3T3 + Y culture conditions have clear applications in disease modeling in respiratory conditions with airway epithelial involvement, further work is required to realize its applications in modeling human cancer and in the development of personalized cancer therapies.
